# Abiraterone and Galeterone, Powerful Tools Against Prostate Cancer: Present and Perspective

**DOI:** 10.3390/pharmaceutics16111401

**Published:** 2024-10-30

**Authors:** Ivana Z. Kuzminac, Andrea R. Nikolić, Marina P. Savić, Jovana J. Ajduković

**Affiliations:** Department of Chemistry, Biochemistry and Environmental Protection, Faculty of Sciences, University of Novi Sad, Trg Dositeja Obradovića 3, 21000 Novi Sad, Serbia; ivana.kuzminac@dh.uns.ac.rs (I.Z.K.); andrea.nikolic@dh.uns.ac.rs (A.R.N.); jovana.ajdukovic@dh.uns.ac.rs (J.J.A.)

**Keywords:** abiraterone, galeterone, VN/124-1, TOK-001, synthesis, cancer, bioactivity, side effects, CYP17A1

## Abstract

Due to the high prostate cancer incidence worldwide, the development of different methods of treatment continues to be a hot research topic. Since its first clinical application at the beginning of the 2010s, abiraterone in the form of prodrug abiraterone acetate continues to be the most used hormone derivative in the treatment of castration-resistant prostate cancer. This is the reason behind the publication of many scientific results regarding its synthesis, biological activity, metabolism, novel designed steroid derivatives based on its structure, etc. A similar steroid compound with a heterocycle in the C17 position, called galeterone, also designed to treat prostate cancer, continues to be in clinical studies, which provides further proof of the importance of these steroid derivatives. Besides prostate cancer treatment, abiraterone showed indications for possible clinical application in the treatment of breast, ovarian, lung, kidney, salivary gland, and adrenocortical cancer, congenital adrenal hyperplasia, Cushing’s syndrome, and COVID-19, while galeterone is investigated for its use against prostate, pancreatic, and breast cancer. Herein, we report a review comprising methods of synthesis, possible clinical applications, and mechanisms of action, as well as structures and bioactivities of derivatives of these two important steroids.

## 1. Introduction

**Figure 1:**
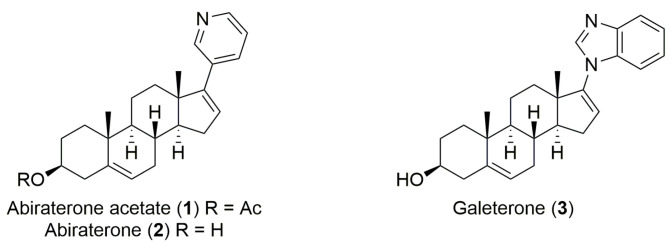
Structures of abiraterone acetate (**1**), abiraterone (**2**), and galeterone (**3**).

Prostate cancer (PC) is the fourth cancer by incidence worldwide, the second most frequent cancer in men, and the fifth leading cause of cancer death among men in 2022. It is the most frequently diagnosed cancer among men in 118 countries (out of 185) [1]. This led to the development of a number of different PC treatment methods, such as surgery, radiation therapy, hormone therapy, chemotherapy, targeted therapy, immunotherapy, and bisphosphonate therapy, and some new types of treatment are being tested in clinical trials [2]. Hormone therapy is essential because PC development and growth are often associated with androgens due to the presence of androgen receptors in cancer cells. Huggins and Hodges first discovered this connection between androgens and PC in the early 1940s, and their orchiectomy application in PC treatment marks the beginning of androgen deprivation therapy (ADT) [3]. ADT has two mechanisms of action: preventing androgen from binding to androgen receptors using antiandrogens, and reducing androgen concentrations by preventing their biosynthesis. The second one is especially important in castration-resistant prostate cancer (CRPC), where residual androgens, synthesized by adrenal glands and intratumors, prevail [4]. The most well-known drug with this mechanism of action is abiraterone acetate (AA, **1**, Figure 1), also known under its IUPAC name as 17-(pyridin-3-yl)androsta-5,16-dien-3β-yl acetate, as well as trade names Zytiga and Yonsa [5]. Its active metabolite abiraterone (**2**, Figure 1) is a potent, irreversible, and selective inhibitor of 17α-hydroxylase/C_17,20_-lyase (CYP17A1), an enzyme involved in androgen biosynthesis in testicular, adrenal, and prostatic tumor cells. AA was developed as an orally bioavailable prodrug with enhanced stability and absorption, because of abiraterone’s poor oral bioavailability [6]. These two compounds were synthesized at the beginning of the 1990s by Barrie and colleagues [7], while abiraterone acetate was approved for clinical use in 2011 [8]. Its usage continues to date, primarily for the treatment of metastatic castration-resistant prostate cancer and metastatic high-risk castration-sensitive prostate cancer. Nevertheless, a search for novel hormone therapeutics in PC treatment never stops, and one of the compounds that have progressed the furthest in clinical studies is galeterone (**3**, Figure 1). Similar to abiraterone, it has a nitrogen-containing heterocyclic ring in the side chain; its IUPAC name is 17-(1*H*-benzimidazol-1-yl)androsta-5,16-dien-3β-ol and it has a triple mechanism of action, acting as an androgen receptor antagonist, androgen receptor down-regulator, and CYP17A1 inhibitor. Galeterone synthesis was first published in 2005 by Handratta et al. [9], and since then, clinical studies have been canceled and continued multiple times. The importance of abiraterone in PC treatment as well as galeterone in drug development motivated us to prepare this review that covers literature analysis regarding the synthesis of these two compounds, recent advances in their biological activity, and structural modifications that could lead to improved anticancer agents.

## 2. Literature Research Analysis

**Figure 2:**
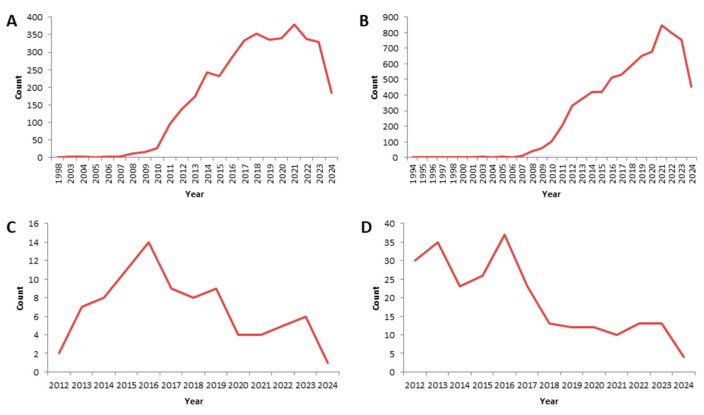
Number of published papers by year from PubMed (results obtained using the “All” search field and keywords “abiraterone” (**A**) and “galeterone” (**C**)) and Scopus (results obtained using the “Article title, Abstract, Keywords” field and keywords “abiraterone” (**B**) and “galeterone” (**D**)).

Six online databases, Scopus, PubMed, Web of Science, SciFinder, ScienceDirect, and Google Scholar, were used during August and September of 2024 to retrieve studies in the literature regarding abiraterone and galeterone. Two primary searches were performed using “abiraterone” and “galeterone” as search terms on 16 August 2024. A search within all fields regarding abiraterone provided 19,864 results in Scopus, 3553 in PubMed, 6372 in Web of Science, 6675 in ScienceDirect, and 45,600 in Google Scholar. As expected, the search term “galeterone” gave significantly lower numbers, with 712 results in Scopus, 90 in PubMed, 124 in Web of Science, 256 in ScienceDirect, and 1240 in Google Scholar, still indicating a need for more refined searches. Hence, the literature search was carried out within the “Article title, Abstract, Keywords”, “Title, Abstract”, and “Topic” research fields in Scopus, PubMed, and Web of Science, respectively. Furthermore, refined searches were performed in all databases using additional keywords: synthesis, biotransformation, biological activity, clinical studies, analogs, derivatives, and modifications. Since the first publication of abiraterone synthesis in 1993, publication numbers regarding abiraterone continued to grow in both PubMed and Scopus (Figure 2A,B), while the number of publications regarding galeterone reached its peak in 2016 (Figure 2C,D).

**Figure 3:**
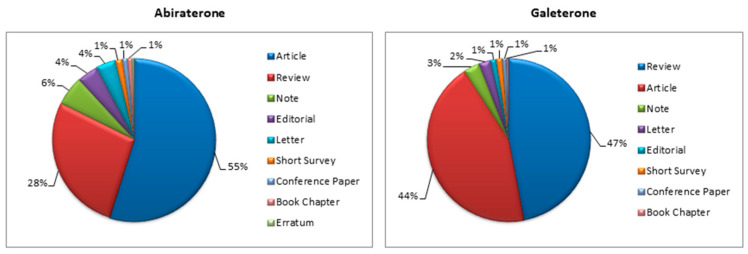
Distribution of documents by type from Scopus (results obtained using keywords “abiraterone” or “galeterone” and the “Article title, Abstract, Keywords” field).

For abiraterone, most of the publications were research articles (55%), while for galeterone, most were review articles (47%, Figure 3). Most of the review articles dedicated to abiraterone involve a comparison of the biological activities of abiraterone acetate and other drugs, such as enzalutamide [10]. There are also reviews dedicated solely to medical applications of abiraterone [11,12], but none of those have the point of view of medicinal chemists and include synthetic procedures and biologically active derivatives. To our knowledge, unlike abiraterone, there is only one review dedicated solely to galeterone [13].

**Figure 4:**
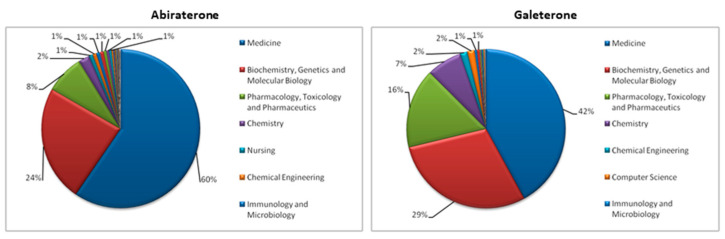
Distribution of documents by subject area from Scopus (results obtained using keywords “abiraterone” or “galeterone” and the “Article title, Abstract, Keywords” field).

It can be observed that the majority of the publications are from the medicinal research field (Figure 4), which shows the distribution of documents by subject area, where fields with the most publications are medicine, biochemistry, genetics and molecular biology, pharmacology, toxicology and pharmaceutics, and chemistry.

**Figure 5:**
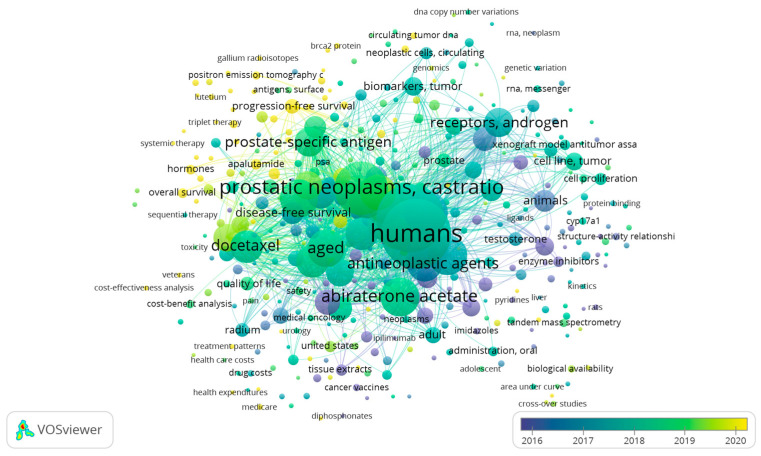
VOSviewer generated an overlay visualization graph with the co-occurrence of keywords related to abiraterone.

**Figure 6:**
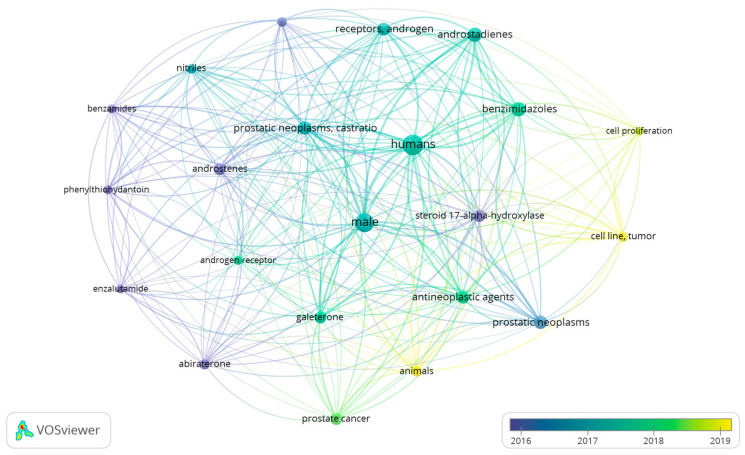
VOSviewer generated an overlay visualization graph with the co-occurrence of keywords related to galeterone.

Maps based on bibliographic data from PubMed (Figure 5 and Figure 6) were created using VOSviewer software (v. 1.6.18, www.vosviewer.com downloaded on 18 November 2022 and used on 16 August 2024).

These maps represent connections between used keywords, and applied analysis used the co-occurrence of keywords, with a minimum number of occurrences set at 10. The number of publications in which the term was found is represented by the size of a keyword and bubble size, and the distance between two keywords offers an approximate relatedness. In addition, overlay visualization gives fast color-coded inside in connection of the keyword and year of publication. For abiraterone, 5225 keywords were detected by PubMed, and 381 met this threshold (Figure 5). The most frequent keywords were “human”, “abiraterone acetete”, “prostatic neoplasms”, and “castration”, while the newest keywords were “progression-free”, “survival”, “apalutamide”, “systemic therapy”, etc. A search for galeterone gave 425 keywords and only 22 met the threshold set at 10 (Figure 6). In addition to “human”, “prostatic neoplasms”, and “castration”, the most frequent keywords were “androstadienes”, “receptors, androgen”, and “benzimidazoles”, while the newest keywords were “cell proliferation”, “cell line, tumor”, and “animals”.

Since abiraterone was synthesized 10 years prior to galeterone and has been in clinical use for more than 10 years, there are 30 to 50 times more publications (depending on the database) regarding abiraterone than galeterone. The number of publications involving abiraterone continues to grow since this compound is in clinical use, unlike galeterone, which showed a decrease in publications because it is still in development. For the same reasons, galeterone has a balance in publications regarding medicine and other research fields, unlike abiraterone, which no longer shows a need for research outside medical applications. Publications regarding these two compounds possess the same three keywords that have the most frequent appearance, but since galeterone is still in development, abiraterone has a higher percentage of research articles (55%, while galeterone has 44%).

## 3. Synthesis

### 3.1. Synthesis of Abiraterone (***2***)

**Scheme 1:**
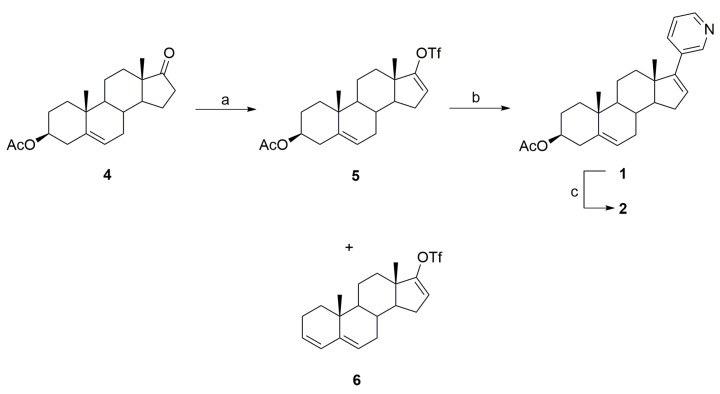
Chemical transformation of dehydroepiandrosterone 3-acetate (**4**) to abiraterone (**2**). Reagents and reaction conditions: (a) Tf_2_O and DTBMP; (b) 3-PyBEt_2_, Pd(PPh_3_)_2_Cl_2_, THF, H_2_O, and Na_2_CO_3_; (c) NaOH, H_2_O, and MeOH.

Although the syntheses of steroids having a pyridin-3-yl or 3-pyridonyl group in the 17β position are already known [14,15], Barrie et al. [7] synthesized a series of steroidal compounds which contain, in addition to a 17-(pyridin-3-yl) substituent, a 16,17-double bond in ring D. This led to the development of 17-(pyridin-3-yl)androsta-5,16-dien-3β-ol (**2**), which was identified as a potent inhibitor of CYP17A1 [16]. Starting dehydroepiandrosterone 3-acetate (**4**) was converted into 17-enol triflate **5** by treatment with trifluoromethanesulfonic anhydride and 2,6-di-*tert*-butyl-4-methyl pyridine (DTBMP) (Scheme 1). This reaction also produced 3,5-diene **6** in 10% yield as a by-product. Then, the pyridin-3-yl group was introduced into the C17 position in the reaction of compound **5** with diethyl(pyridin-3-yl)borane (BET) in THF, using bis(triphenylphosphine)palladium(II) chloride as a catalyst and aqueous Na_2_CO_3_ as a nucleophilic activator. The acetyl group of compound **1** was removed with aqueous methanolic NaOH to produce steroid **2** [7,17].

Since the reported syntheses [17,18,19] of triflate **5** used DTBMP as a base, which is expensive and leads to the formation of by-product **6**, it was necessary to improve the synthesis. To improve the procedure for obtaining the key intermediate triflate **5**, tertiary or heterocyclic amines were selected as bases for the reaction, so the pKa of the conjugate acid at 25 °C is within the range 5.21 to 12 [20]. The most preferable base was 2,6-lutidine or triethylamine. To minimize the decomposition of the product, it was suggested that the base should be added to the reaction mixture shortly after triflic anhydride, and the reaction mixture should be quenched within an hour after the addition of the base. However, in this procedure, in addition to the formation of **5**, there was also starting compound **4**. According to the authors, this method of synthesis allows for avoiding chromatographic purification, and to obtain pure abiraterone (**2**), it was suggested to convert it into salt with methanesulfonic acid, and then recrystallize the obtained salt from isopropanol and thus separate **2** from starting compound **4** [20,21].

In the next procedure for the synthesis of key triflate **5**, dehydroepiandrosterone 3-acetate (**4**) reacted with a trifluoromethanesulfonic anhydride, in an inert solvent in the absence of an organic base or the presence of an inorganic base, preferably sodium or potassium carbonate [22]. The crude triflate may be purified by crystallization from a suitable solvent: aliphatic alcohol (e.g., methanol or isopropanol), acetic acid, acetic anhydride, or any mixture thereof with water.

Sun et al. [23] reported that the triflation reaction carried out in tetrahydrofuran (THF) at −78 °C in the presence of PhN(Tf)_2_ and potassium hexamethyldisilazane gives better results than the reaction with triflic anhydride in methylene chloride with NaH or pyridine. However, the experimental results show that under these conditions, an improvement in the triflation reaction was achieved, but the obtained abiraterone acetate (**1**) also contains a by-product of 17-(*N*-phenylamino)androsta-5,16-dien-3β-yl acetate that cannot be removed [24].

Bian et al. [25] developed an efficient eco-friendly method for the synthesis of AA (**1**) and analogs via a green Suzuki reaction in water-PEG-400 at 75 °C from various arylboronic acids and 17-(trifluoromethanesulfonyl)oxy-3β-acetoxylandrosta-5,16-diene (**5**). According to the reported method [17], compound **5** was prepared by treating compound **4** with trifluoromethanesulfonic anhydride and dry triethylamine in dry CH_2_Cl_2_ at 0 °C and separating them by column chromatography.

**Scheme 2:**
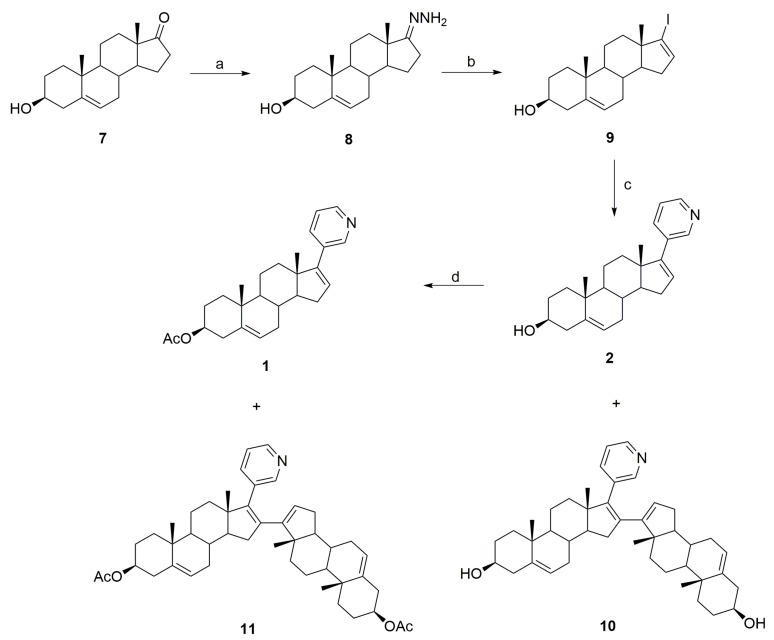
Synthesis of **1** from **7** via 17-hydrazone **8**. Reagents and reaction conditions: (a) 1. N_2_H_4_xH_2_O; 2. N_2_H_4_ x H_2_SO_4_, EtOH; (b) 1. I_2_, THF; 2. TMG; (c) 3-PyBEt_2_, Pd(PPh_3_)_2_Cl_2_, THF, Na_2_CO_3_, H_2_O; (d) Ac_2_O, Py.

Potter et al. [26] have modified the classical synthesis of abiraterone acetate (**1**). As previously mentioned, the classical method is not commercially viable due to the usage of expensive and noxious triflic anhydride. The improved method involved the synthesis of vinyl iodide **9** which is easily and cheaply obtained via the corresponding 17-hydrazone **8** by iodine oxidation in the presence of a hindered guanidine base, 1,1,3,3-tetramethyl guanidine (TMG), in the yield of 95% (Scheme 2). The palladium-catalyzed cross-coupling reaction of **9** with BET proceeded without the need to protect the 3-hydroxyl function, yielding abiraterone (**2**).

The disadvantage of this method is the long cross-coupling reaction time (4 days at 80 °C) which leads to the formation of by-product **10**. Compound **10** can be reduced either by keeping the proportion of borane to 1.2 equivalents according to steroids or by crystallizing the reaction product of the cross-coupling reaction from a mixture of acetonitrile and methanol. Acetylated form **11** can be separated from AA (**1**) only by reverse-phase chromatography.

For the synthesis of **1**, Negashi coupling has also been reported [27]. The reaction of iodine **9** with pyridyl zinc halide in the presence of a palladium catalyst followed by acetylation afforded the synthesis of AA (**1**). The preparation of pyridyl zinc halide from 3-bromopyridine in the presence of *n*-butyl lithium involves extensive precautions and is not recommended for commercial-scale usage.

In the next report, abiraterone acetate (**1**) was produced by the four-step synthesis with 43.5% overall yield and 99.8% purity, without using chromatographic separation in the final step [28]. In the modified procedure for obtaining hydrazone 8, H_2_SO_4_ was used as a catalyst and the yield was 96%. Several factors had an impact on the duration of the Suzuki cross-coupling reaction, the yield, and the purity of **1**. These included the amount of the catalyst, the base, and the solvent. A series of experiments showed that the optimum system for the reaction was dioxane–H_2_O as the solvent and Na_2_CO_3_ as the base. Increasing the amount of catalysts shortened the reaction time.

**Scheme 3:**
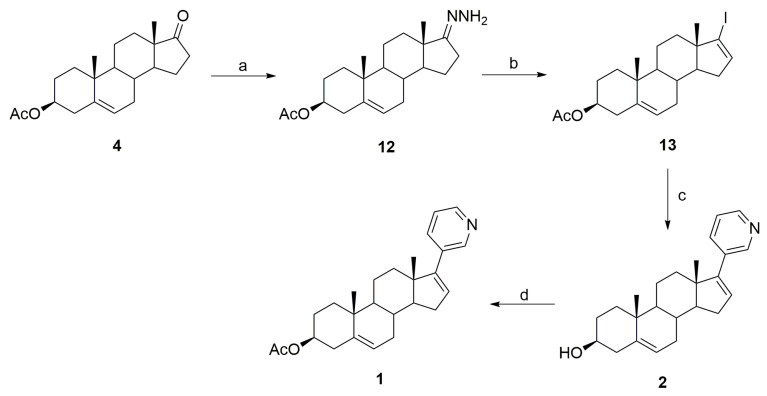
Synthesis of **1** from **4** via 17-hydrazone **12**. Reagents and reaction conditions: (a) N_2_H_4_xH_2_O, EtOH, AcOH; (b) 1. I_2_, THF; 2. TMG; (c) 1. 3-PyBEt_2_, Pd(PPh_3_)_2_Cl_2_, THF, Na_2_CO_3_, Bu_4_N^+^F^−^; 2. MeOH, NaOH; (d) Ac_2_O, DMAP, CH_2_Cl_2_.

Liu et al. [29] studied the reaction conditions for hydrazone synthesis, an iodine reaction, cross-coupling, and acetylation, to reduce the costs, avoid the formation of by-products, and make the route suitable for large-scale production. The best molar ratio of **4** and hydrazine hydrate in the synthesis of hydrazone **12** was 1.42–1.50. The solvent of choice was absolute ethanol, its weight ratio was 5, and at the same time, CH_3_COOH was added, at a molar ratio of 0.18. The yield of **13**, when the molar ratio of compound **12**, iodine, and TMG were 1.95 and 3.80, respectively, was about 75%. The route to commercially available AA (**1**) was obtained with a 52% total yield (Scheme 3).

Komati et al. [30] optimized methods for obtaining compound **1** via hydrazone and vinyl iodide. They achieved 95, 85, and 82% selectivity and 99, 96, and 99% purity of hydrazone **12**, vinyl iodide **13**, and final compound **1**, respectively. The overall yield increased to 57%.

**Scheme 4:**
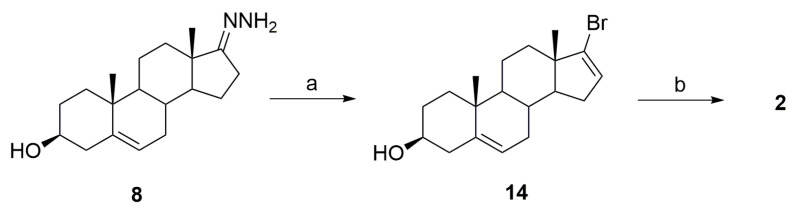
Synthesis of **2** from **8** via 17-bromo derivative **14**. Reagents and reaction conditions: (a) NBS, TMG; (b) 3-PyBEt_2_, Pd(PPh_3_)_4_, Na_2_CO_3_, *t*-BuOH.

In order to overcome the shortcomings of the reported methods of synthesis of abiraterone (**2**), Mahra et al. [31] used 17-bromoandrosta-5,16-dien-3β-ol (**14**) instead of 17-iodo derivative **9** for Suzuki coupling (Scheme 4). Due to the shorter reaction time, bis-steroid **10** was not formed.

**Scheme 5:**
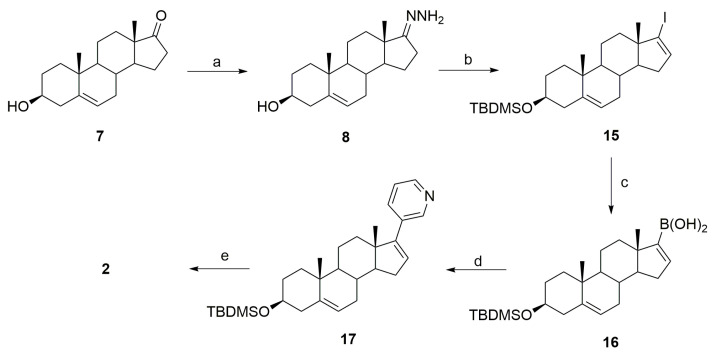
Synthesis of **2** from **7** via boronic acid of 3-silyl-protected dehydroepiandrosterone **16**. Reagents and reaction conditions: (a) NH_2_NH_2_xH_2_O; (b) 1. Iodine, THF; 2. TBDMS-Cl; (c) THF, *n*-BuLi, B(OEt)_3_; (d) 3-bromopyridine, Pd catalyst, Na_2_CO_3_; (e) Bu_4_N^+^F^−^, THF.

In another reported method, boronic acid of 3-silyl-protected dehydroepiandrosterone **16** was coupled with 3-bromopyridine and resulted in **17** (Scheme 5) [32]. After deprotection, abiraterone (**2**) was obtained. The drawbacks of this method are the use of protective groups and the need for chromatographic purification.

**Scheme 6:**
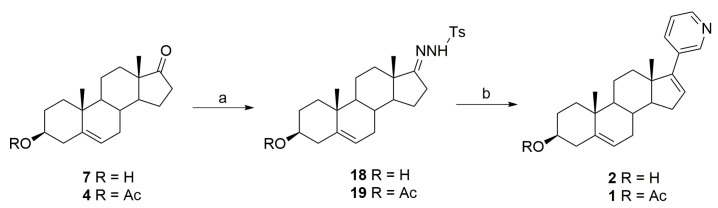
Synthesis of **1** and **2** from **4** and **7** via tosylhydrazones **19** and **18**. Reagents and reaction conditions: (a) NH_2_NHTs, TsOH; (b) 3-brompyridine, catalyst, *t*-BuOLi.

The same authors [32] reported obtaining compounds **1** and **2** by C-C coupling via steroid hydrazone, such as tosylhydrazones **19** and **18** (Scheme 6), but with lower yields.

Li et al. [33] reported the synthesis of abiraterone acetate (**1**) from dehydroepiandrosterone **7** via formation of tosylhydrazone, a cross-coupling reaction, and acetylation, including column chromatography and recrystallization with an overall yield of 51.9%.

**Scheme 7:**
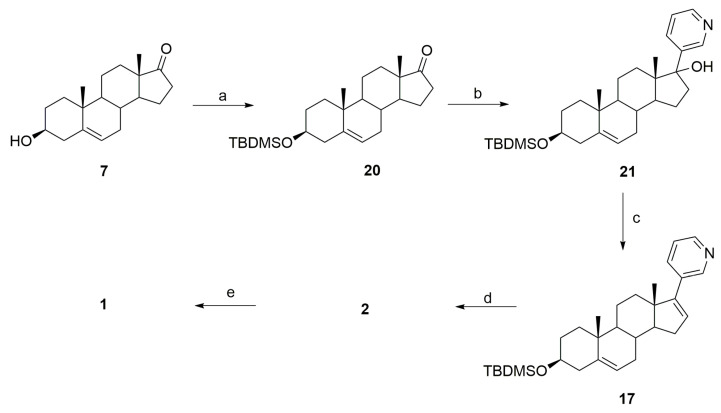
Synthesis of **1** from **7** via pyridinyl hydroxyl derivative **21**. Reagents and reaction conditions: (a) TBDMS-Cl, CH_2_Cl_2_, imidazole; (b) 3-bromopyridine, dry toluene/THF, *n*-BuLi; (c) MsCl, TEA, CH_2_Cl_2_; (d) TBAF, THF; (e) Ac_2_O, Py.

Marom et al. [34] reported a new method with mild reaction conditions and low-cost reagents for abiraterone acetate (**1**) production. The process includes coupling of protected dehydroepiandrosterone derivative **20** with 3-bromopyridine to give pyridinyl hydroxyl derivative **21**, then dehydration of **21** and transformation of **17** into **1** (Scheme 7).

### 3.2. Synthesis of Galeterone (***3***)

**Scheme 8:**
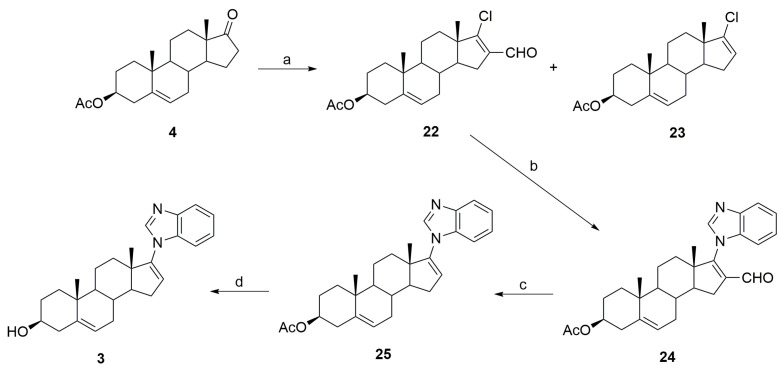
Synthesis of galeterone (**3**) from dehydroepiandrosterone acetate (**4**). Reagents and conditions: (a) POCl_3_-DMF, CH_3_Cl, Ar, reflux; (b) benzimidazole, K_2_CO_3_, DMF, Ar, 80 °C; (c) 10% Pd on activated charcoal, PhCN, reflux; (d) 10% methanolic KOH, Ar, rt.

Although the structure of galeterone (**3**) was published in 2003 [35], its synthesis was first reported by Handratta et al. [9] in 2005. The key intermediate in the synthesis, 3β-acetoxy-17-chloro-16-formylandrosta-5,16-diene (**22**), was obtained in the reaction of **4** with phosphorus oxychloride (POCl_3_) and dimethylformamide (DMF) with 77% yield. In addition, the 16-deformylated compound **23** was obtained as a side product with 11.4% yield (Scheme 8) [36,37]. Treatment of **22** with benzimidazole in the presence of K_2_CO_3_ in DMF at 80 °C gave compound **24**, which was deformylated with 10% palladium on activated charcoal in refluxing benzonitrile. The obtained compound **25** yielded galeterone (**3**) by hydrolysis. Barbieri et al. [38] further optimized the described method of synthesis of galeterone (**3**).

Purushottamachar et al. [39] reported a large-scale synthesis of galeterone (**3**) from compound **4**, achieving a yield of 59%. Optimization of solvents and reagents during the first two steps allowed for the isolation of compounds **22** and **24** by heptane triturations, eliminating the need for column chromatography. In the deformylation step, the catalyst usage was reduced from 50% to 10% (wt/wt) of compound **24**, simplifying the purification of compound **25** and modestly increasing the yield. Hydrolysis of the C3-acetoxy group of **25** with potassium hydroxide in methanol, at ice bath temperatures, does not involve chromatographic purification.

## 4. Medicinal Uses, Benefits, and Side Effects

### 4.1. Abiraterone (***2***)

#### 4.1.1. Medical Uses and Benefits of Abiraterone

In the middle of the second decade of the 21st century, an expansion of works on abiraterone and its biological activity was noted. Initially, pharmaceutical companies were not interested in clinical trials and production of abiraterone and its 3β-acetate form, although the synthesis was published in the early 1990s [7,16,40]. Interest in abiraterone rose again when prostate cancer was found to be almost completely dependent on male sex hormones, and the first clinical trial paper was published in 2004 [41]. The rights to develop the drug were licensed to the pharmaceutical company Cougar Biotherapeutics (now part of Janssen Pharmaceuticals), and it was officially approved in Europe in 2011, as well as in the USA by the US Food and Drug Administration (FDA), while The National Institute of Health and Care Research approved its use in the United Kingdom by Care Excellence (NICE) in 2012.

Abiraterone acetate is administered orally in tablet form or film-coated tablet form and contains micronized crystalline AA with a diameter within the range of 3–10 µm [42]. However, because this abiraterone acetate formulation is poorly absorbed and exhibits high pharmacokinetic variability in abiraterone exposure, innovative formulations, such as Abiraterone Acetate Fine Particle (AAFP) (using SoluMatrix Fine Particle Technology™), have been investigated. AAFP is designed to increase oral bioavailability, and 500 mg of AAFP has been found to be bioequivalent to the 1000 mg originator formulation of abiraterone acetate [43]. This research has resulted in a newer advanced micronized formulation, approved by the FDA in 2018, where the particle size of abiraterone acetate has been reduced to 200–800 nanometers in diameter (Sun Pharmaceutical Industries, Inc.; Goreagon, Mumbai, India).

**Figure 7:**
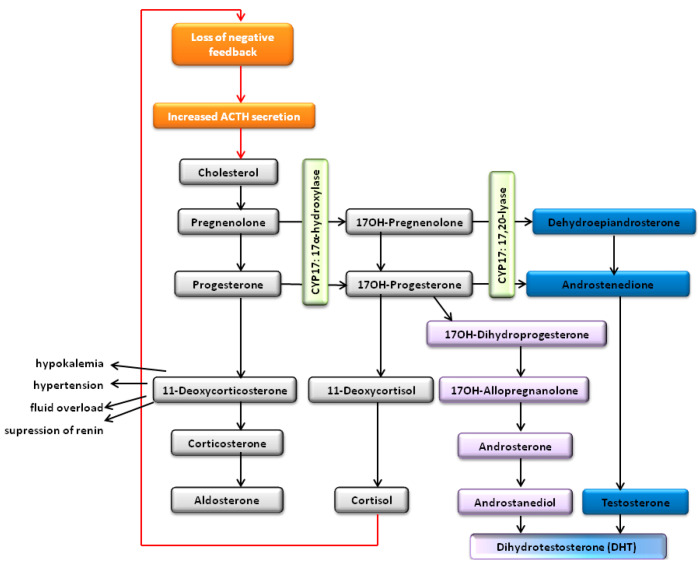
One simplified part of the biosynthesis of androgen hormones with the site of action of the CYP17A1 enzyme, as well as the indicated side effects of abiraterone acetate administration. The classical pathway of androgen synthesis through dehydroepiandrosterone is shown in blue, and an alternative pathway to dihydrotestosterone from 17-hydroxyprogesterone via androsterone is shown in purple (backdoor pathway).

Human cytochrome P450 17A1 (CYP17A1) plays a critical role in the production of glucocorticoids and sex hormones and has been implicated in a number of disease states. Cytochrome P450 17A1 is an enzyme that catalyzes the activities of both 17α-hydroxylase and 17,20-lyase, mainly in the adrenal glands and gonads, encoded by a single gene formally called *CYP17A1*. Human 17α-hydroxylase/17,20-lyase hydroxylates both pregnenolone and progesterone with approximately equal efficiency, while 17,20-lyase activity is about 50 times more efficient for the conversion of 17-hydroxypregnenolone to dehydroepiandrosterone (DHEA) than for the conversion of 17-hydroxyprogesterone to androstenedione (Figure 7) [44,45]. The discovery of the role of CYP17A1 in androgen biosynthesis in humans suggests that its inhibition is an excellent strategy for preventing androgen synthesis and treating androgen-dependent diseases. X-ray crystal structures of CYP17A1*,* obtained in the presence of abiraterone or galeterone, clarify their mechanism of action, where both inhibitors bind heme iron, which can further contribute to the development of inhibitors that exclusively inhibit cytochrome P450 17A1 lyase activity. Structures of CYP17A1 with abiraterone and galeterone show a characteristic cytochrome P450 fold. Heme iron binds nitrogen from the pyridine or benzimidazole core, from abiraterone or galeterone, respectively, via a coordinate covalent bond. The steroid core is oriented between the F and G helices and rises at an angle of 60° above the heme. Furthermore, the steroid molecule is oriented with its less shielded α-side towards helix I where Gly301, Ala302, and adjacent residues form a highly complementary hydrophobic flat surface. The 3β-OH group of the steroid molecule form a hydrogen bond with Asn202 in the F helix. The hydrogen bond network also includes Glu305, several conserved water molecules, Arg239, the carbonyl of Gly297, and, in some cases, Tyr201. Similar binding was observed with galeterone [46].

With the discovery of the biological activity of abiraterone, after its synthesis, most of the published articles focused on prostate cancer. After extensive work, clinical trials, and effective use in the treatment of prostate cancer, scientists have focused on studying the effects of abiraterone in other cancers where androgen dependence has been observed.

##### Prostate Cancer

Prostate cancer shows androgen dependence in most cases, so surgical or medical castration in the initial stages is used to treat this disease. With long-acting gonadotropin-releasing hormone agonists or antagonists, which lead to testicular suppression, remission of the disease can be achieved [47]. But often, despite therapy, progression occurs, due to traces of residual androgens, and such a condition is described as castration-resistant prostate cancer (CRPC) [48].

The androgen receptor (AR) is known to play a key role in the pathogenesis of prostate cancer and can be activated by ligand testosterone or 5α-dihydrotestosterone (DHT). Therefore, the therapeutic strategy of androgen deprivation therapy (ADT) is rational and useful in the treatment of prostate cancer. On the other hand, intratumoral secretion of enzymes involved in testosterone synthesis, such as CYP17A1, supports tumor survival and growth. Understanding these molecular mechanisms has led to the development of newer drugs that work by inhibiting androgen-producing enzymes or blocking the AR, called androgen receptor signaling inhibitors (ARSIs) [49]. Among them is abiraterone. As already mentioned, O’Donnell et al. [41] published the first report on the use of a specific CYP17A1 inhibitor in humans. Renewed interest and expansion of works related to the biological activity of abiraterone appeared about 10 years after the publication of the synthesis of abiraterone and abiraterone acetate [50,51,52,53,54], which led to its approval and application in the treatment of prostate cancer.

In phase III clinical studies, AA combined with prednisone was shown to prolong overall survival in metastatic CRPC, with an acceptable toxicity profile [55], although some studies confirmed that after treatment with abiraterone acetate for metastatic castration-resistant prostate cancer (mCRPC), retreatment with AA is associated with limited clinical benefit [56]. On the other hand, in newly diagnosed patients with high-risk metastatic castration-sensitive prostate cancer (mCSPC), some recent studies confirmed the benefits of using abiraterone acetate in combination with prednisone as ADT [57].

More recent studies of dual pathway inhibition with the AKT inhibitor ipatasertib plus abiraterone indicate that this therapy may have an improved effect over abiraterone alone. Abnormal serine/threonine kinase activity has been observed in cancer and it plays an important role in the Akt (protein kinase B) phosphatidylinositol 3-kinase (PI3K)/Akt/mammalian target of the rapamycin (mTOR) signaling pathway. The AKT inhibitor ipatasertib in combination with abiraterone and prednisolone has been used in Japanese patients with advanced or recurrent refractory solid tumors, and currently, there are ongoing global phase III studies for ipatasertib and abiraterone [58,59,60].

Polyadenosine diphosphate-ribose polymerase (PARP) inhibitors have shown significant activity in patients with various cancers, with the greatest clinical benefit for BRCA1/2 mutation carriers. Niraparib, an orally bioavailable, potent, and highly selective inhibitor of PARP-1 and PARP-2, is currently being investigated for the treatment of metastatic prostate cancer. In the phase 3 MAGNITUDE study, niraparib in combination with AA plus prednisone (AAP) was confirmed to have clinical benefit in patients with BRCA1/2-altered mCRPC [61,62,63]. On the other hand, low-dose abiraterone plus olaparib, another PARP inhibitor, might be an alternative late-line treatment with a manageable safety profile for selected patients with mCPRC who had no mutations of BRCA1/2 [64].

##### Breast Cancer

The term triple-negative breast cancer (TNBC) is defined by negative clinical testing for three receptors: estrogen receptor (ER), progesterone receptor (PR), and human epidermal growth factor receptor 2 (HER2) amplification [65]. Because it lacks these receptors, TNBC does not respond to conventional targeted therapies for breast cancer such as endocrine therapy or HER2-targeted therapies, leaving only chemotherapy as an option. Further molecular and immunohistochemical analyses show that a subset of TNBCs expresses the androgen receptor (AR) [66]. In their research, Barton et al. [67] suggested that AR testing should become the standard in tumor analysis and that TNBC should be defined as either AR+ TNBC or “quadruple negative” disease to highlight the presence of AR as a potential target for the design and application of new drugs. In their research, Fioretti et al. [68] described AR as a useful marker and therapeutic target for the management of breast cancer (BC), but its impact on prognosis and its predictive role in TNBC are still controversial. Since a subset of triple-negative BC has been shown to depend on androgen signaling for growth, therapies that inhibit androgen signaling have undergone clinical trials, including abiraterone acetate. In clinical trials, abiraterone acetate was investigated as a single therapy or in combination with other drugs.

In phase I clinical trials, 25 patients were treated with AA. Basu et al. [66,69] concluded that AA was well tolerated in patients with advanced breast cancer, with preliminary evidence of antitumor activity.

In their research, Bonnefoi et al. [70] performed a phase II trial in women with ER-negative and AR-positive (molecular apocrine-like HER2-negative) metastatic or inoperable locally advanced BC with the aim of assessing the efficacy and safety of AA plus prednisone. Based on their research, AA treatment with prednisone was found to be beneficial for some patients. Prednisone is prescribed to prevent secondary mineralocorticoid excess syndrome and may stabilize tumor growth or help maximize the effect of AA on its own.

Grellety et al. [71] described a case report, in which they concluded within phase II that abiraterone has been shown to improve the sensitivity of TNBC cell lines toward immune-mediated lysis. In their further clinical trial in patients with AR-positive TNBC, Grellety et al. proposed abiraterone acetate combined with a Chk1 inhibitor, and the sensitization of cells with a Chk1 inhibitor might be an innovative therapeutic strategy. They proposed Chk1, a protein serine/threonine kinase essential for the maintenance of genomic integrity, as a potential novel target in AR-positive TNBC [72].

Rhanine et al. [73] conducted a study on the real-life clinical benefit rate of antiandrogens in patients with AR-positive TNBC between 2013 and 2020. A standardized questionnaire was sent to 30 centers in France, where of 24 patients, 62% used abiraterone acetate in therapy. Their real-life data were consistent with findings from previously published clinical trials, with overall limited antitumor activity but with a significant subset of patients experiencing clinically relevant benefits.

While androgens are precursors for estrogen production, it was further hypothesized that abiraterone could be an effective form of treatment for ER-positive breast cancer.

Ng et al. [74] reported a phase I/II trial of abiraterone acetate in ERα or AR-positive metastatic breast cancer (MBC). Their phase I/II trial of AA with hydrocortisone evaluated tolerability, pharmacokinetics, pharmacodynamic profiles, and antitumor activity. The authors concluded that AA was well tolerated and merits further evaluation in MBC.

Capper et al. [75] investigated whether abiraterone could bind to ER and have estrogenic activity in ER-positive MCF-7 and T47D breast cancer cell lines. In their work, the authors found that abiraterone directly activates the ER. Because abiraterone induces cell proliferation and activates ER, the authors suggest that abiraterone should be combined with other ER antagonists when used for the clinical treatment of ER-positive breast cancer.

In their study, O’Shaughnessy et al. [76] examined whether combined inhibition of androgen biosynthesis with abiraterone acetate plus prednisone and inhibition of estradiol synthesis with exemestane could be of clinical benefit in ER-positive postmenopausal patients previously treated with nonsteroidal aromatase inhibitors (NSAIDs). The addition of abiraterone acetate to exemestane in NSAID-pretreated ER+ metastatic breast cancer patients did not improve progression-free survival compared with treatment with exemestane alone. The authors believe that the abiraterone acetate-induced increase in progesterone may have contributed to this lack of clinical activity [76,77].

Similarly, Simigdala et al. [78] investigated the effect of abiraterone on ER signaling in a panel of ER+ breast cancer cell lines (MCF7, HCC1428, and SUM44) sensitive or resistant to long-term estrogen deprivation, modeling relapse on an aromatase inhibitor and containing different natural estrogen receptor 1 (ESR1) mutations. Their data suggest that abiraterone may have a context-dependent role in ER+ breast cancer that may be influenced by prior hormone therapy and that ESR1 mutational status may influence its efficacy in the clinical setting. The study also confirms the estrogenic activity of abiraterone.

The ability of abiraterone to improve the sensitivity of breast cancer cells to immune-mediated lysis was studied by Kwilas et al. [79] on two breast cancer cell lines: AR-positive luminal B (ZR75-1), and ER- and AR-negative mesenchymal stem-like cell lines (MDA-MB-231). Abiraterone increased the sensitivity of both AR-positive and AR-negative breast cancer cells to cytotoxic T lymphocyte-mediated lysis. These studies confirm the ability of abiraterone to increase the sensitivity of breast cancer cells to immune-mediated killing regardless of AR status. Therefore, the use of AR inhibition is a potential new therapeutic option for AR-positive TNBC and for the treatment of AR-negative TNBC, especially in combination with cancer immunotherapy.

In conclusion, it can be pointed out that abiraterone acetate represents a suitable therapy for the treatment of breast cancer, in combination with other therapies. Particularly, abiraterone should be combined with other ER antagonists in the clinical treatment of ER-positive breast cancer, but an individualized approach to each patient is necessary in order to select the appropriate therapy.

##### Ovarian Cancer

In epithelial ovarian cancer (EOC), AR is expressed more often than ER and has been reported to be detectable in up to 90% of cases [80]. Papadatos-Pastos et al. [81] described the association of increased hyperandrogenism with a higher risk of ovarian cancer, but therapeutic approaches that inhibit androgen signaling have produced only modest response rates. In light of new data, regarding the role of androgen stimulation in the evolution of EOC and the emergence of new compounds used to treat other hormone-induced malignancies, abiraterone has been suggested as a potential biomarker [80]. However, clinical studies on the use of abiraterone are quite rare [82].

The Cancer of the Ovary Abiraterone trial (CORAL) was designed to investigate AR-targeted agents in ovarian cancer. The objective of the study was to evaluate the activity and safety of AA plus prednisone in hormone-naïve patients with recurrent epithelial ovarian, fallopian tube, or primary peritoneal cancer. Responses were rare, but a subset of patients achieved sustained clinical benefit. Although AA was found to significantly reduce estradiol and testosterone levels, the AR signaling pathway is not the most critical driver of tumor progression in recurrent EOC, and the authors concluded, similar to breast tumors [75], that the preferred therapy is a combination of CYP17A1 inhibitors (abiraterone) with drugs such as the AR antagonist enzalutamide or fulvestrant [83].

##### Congenital Adrenal Hyperplasia

Congenital adrenal hyperplasia (CAH) is an autosomal recessive disorder that affects the cortex of the adrenal gland and is caused by a deficiency of the enzyme 21-hydroxylase. Inadequate conversion of 17-hydroxyprogesterone to 11-deoxycortisol leads to impaired cortisol synthesis, while 21-hydroxylase deficiency (21-OHD) also causes the incorrect secretion of aldosterone [84,85].

Further, reduced cortisol production through feedback affects the increase in the secretion of the pituitary adrenocorticotropic hormone (ACTH) (Figure 7), which leads to excessive production of adrenal androgens and hyperplasia of the adrenal gland. Patients with 21OHD-CAH have adrenal insufficiency, which can cause life-threatening adrenal crises, while excess androgens cause atypical genitalia in infants, promote abnormal growth, culminate in short adult stature, and sometimes cause precocious puberty, while in adulthood, they cause female virilization and infertility in both sexes; they have increased mortality up to five times that of the healthy population, increased cardiovascular disease risk factors, and metabolic morbidity [86,87].

In patients with congenital adrenal hyperplasia, the only remaining steroidogenic pathways involve the related steroid enzyme CYP17A1. The flow of accumulated cortisol precursors through CYP17A1 generates the androgen excess characteristic of 21OHD. For this reason, abiraterone has recently appeared as a therapy in the treatment of 21-OHD.

Auchus et al. [88] screened 14 adult women with genotype-proven classic 21OHD. In their study, AA was added to stable doses of physiological hydrocortisone and 9α-fludrocortisone acetate to control androgen excess in 21OHD, and they proved that CYP17A1 inhibition is a good method to control androgen excess in classic 21OHD without using supraphysiological doses of hydrocortisone.

In recent years, the importance of the adrenal synthesis of alternative (backdoor path; purple boxes in Figure 7) and 11-oxygenated androgens has attracted attention, while the enzymes involved in the synthesis of androgens via the alternative pathway were discovered in human fetal adrenal glands (HFAs). Melau et al. [89] aimed to examine the effects of the CYP17A1 inhibitor AA on HFA tissue cultured ex vivo. The effects were examined under basal and ACTH-stimulated conditions in tissue from the same fetus and determined by quantification of adrenal steroid secretion in the culture medium. AA treatment resulted in a strong inhibition of CYP17A1, and ACTH stimulation increased the inhibitory effects. Although AA has previously been suggested to inhibit CYP17A1 and CYP21A2 [90,91,92], the observed increase in corticosterone levels after AA treatment in ex vivo-cultured HFA tissue does not support CYP21A2 inhibition.

The wider significance of 11-oxygen androgens in humans has only recently emerged. 11-Ketotestosterone is the predominant circulating androgen during normal and premature adrenarche, in women and men with poorly controlled 21OHD, as well as in postmenopausal women. The purpose of Wright’s study was to determine the effects of AA administration on the 11-oxygenated androgens: 11β-hydroxyandrostenedione and 11-ketosterone, as well as 11-ketoandrostenedione and 11β-hydroxytestosterone [93]. Their data support the hypothesis that AA therapy lowers 11-oxygenated androgens, consistent with extremely tight binding of abiraterone to CYP17A1. They concluded that AA alone and combined with prednisone (AAP) therapies markedly reduce the production of the adrenal-derived 11-oxygenated androgens.

##### Human Adrenocortical Cancer and Cushing’s Syndrome

Cushing’s syndrome is an endocrine disorder resulting from chronic exposure to excessive doses of glucocorticoids and can be caused by an ACTH-producing pituitary adenoma (Cushing’s disease) or a primary functional adrenocortical tumor. Available treatment options include steroidogenesis inhibitors, centrally acting agents, and glucocorticoid receptor antagonists [91,94].

Adrenocortical carcinoma (ACC) is a highly malignant, treatment-resistant, and extremely rare cancer. The key molecular event that contributes to its formation and aggressiveness is the constitutive activation of the Wnt/β-catenin signaling pathway. Since cortisol hypersecretion is known to lead to hypertension, hypokalemia, hyperglycemia, and infection, and the immunosuppressive effects of excess cortisol can additionally promote tumor progression, prompt control of hypersecretion is therefore crucial for effective treatment. Since AA, besides reducing androgen levels, rapidly impairs cortisol synthesis, it could be potentially used in the management of Cushing’s syndrome and in rapid control of cortisol hypersecretion and tumor growth in patients with ACC. In order to examine the antisecretory and antiproliferative effects of AA, the authors used NCI-H295R and SW13 ACC cell lines and human primary ACC cell cultures, as well as immunodeficient mice, whereby the antitumor efficacy was confirmed in vivo in NCI-H295R cells xenografted in immunodeficient mice. In addition to reducing cortisol and androgen secretion, AA increased progesterone production and caused a decrease in cell viability in NCI-H295R cells and primary secreting ACC cultures. Additionally, AA reduced the nuclear accumulation of Wnt/β-catenin in NCI-H295R cells, and its cytotoxic effect was prevented by either blocking PgR or gene silencing. The authors confirmed that progesterone receptor signaling, via negative modulation of the Wnt/β-catenin pathway, contributes to AA inhibition of cell viability in the ACC [95].

In another study, the same authors examined the hormonal effects of abiraterone administration in a patient with Cushing’s syndrome induced by heavily previously treated metastatic ACC. The patient experienced benefits with the use of AA, while hormonal laboratory tests showed a significant reduction in circulating androgen levels and urinary cortisol excretion [96].

In a case report, a novel use of AA in the treatment of malignant ectopic Cushing’s syndrome (EAS) by blocking cortisol synthesis was presented. EAS, secondary to an adrenocorticotropin hormone (ACTH)-releasing neuroendocrine tumor (NET), is a rare diagnosis. Similar to previous research, it was concluded that cortisol inhibition caused by AA can be effectively used in the management of EAS and that its application can be considered as a temporary measure in the treatment of malignant EAS [97].

##### Lung Cancer

Non-small-cell lung cancer (NSCLC) threatens human life globally with high morbidity and mortality, where radiotherapy is one of the most effective methods for the treatment because tumor angiogenesis can be induced by a low dose of radiation. For this reason, the antiangiogenic effect of abiraterone against human vein endothelial cells (HUVECs) incubated with an irradiated medium of lung cancer cells was investigated. HUVECs were incubated in parallel with the culture medium of NSCLC cell line A549, A549 cells treated with abiraterone, A549 cells treated with radiation, and A549 cells treated with abiraterone and radiation. Abiraterone suppresses the angiogenic capacity of endothelial cells induced by irradiated lung cancer cells, but the mechanism of antiangiogenic activity of abiraterone needs to be further investigated [98].

##### Kidney Cancer

Most kidney cancers include renal cell carcinoma (RCC), which remains one of the deadliest cancers of the genitourinary system. Patients with advanced-stage RCC still have poor prognoses, although the introduction of targeted therapies has revolutionized the treatment of metastatic and recurrent RCC (mRCC). Recently, it has been established that androgen receptors are present in higher-stage tumors regardless of gender, and the assumption that AR-positive RCC has intratumoral steroidogenesis and that antiandrogen therapy can lead to tumor suppression has been studied. In their study, Lee et al. [99] injected mice with an AR-positive RCC cell line. When tumors became palpable, surgical castration was performed and intracellular testosterone and dihydrotestosterone levels were measured in the AR-positive human RCC cell lines. Then, mice bearing xenografts were treated with AA. It was confirmed that intratumoral steroidogenesis is a significant source of androgens in AR-positive RCC, that AA contributes to significant tumor suppression, and that the androgen signaling axis is a potential target for intervention in RCC.

##### Chronic Kidney Disease

It is a common phenomenon that chronic kidney disease occurs along with prostate cancer. Francini et al. [100] presented two cases of castration-resistant metastatic prostate cancer and chronic kidney disease. Both patients developed clinical resistance to ADT and were treated with a conventional chemotherapy approach, leading to deterioration of renal function and performance status. Therefore, they underwent treatment with AA and the patients showed improvement in renal function with a good response to the disease.

##### Salivary Gland Carcinoma

Because of the known role of AR as an immunomarker for the diagnosis of salivary gland duct carcinoma (SDC), as well as other non-squamous-cell head and neck cancers (NSCC-HN), the potential use of newer generation antiandrogens, including abiraterone, is investigated [101]. Although ADT is a promising therapeutic option, there are only a few reports on the efficacy of ADT in metastatic or unresectable salivary gland cancer. Some of the first research studies were carried out by the group of Locati et al. [102]. They described two patients in whom abiraterone therapy proved to be effective in the treatment of salivary gland carcinoma (SGC). Further, they examined the activity of AA as a second-line treatment in AR+ patients with SGC and concluded that abiraterone plus a luteinizing hormone-releasing hormone agonist is active and safe as a second-line option in AR-expressing, castration-resistant SGC [103]. Urban et al. [104] also described the case of the use of abiraterone in the treatment of SDC, as well as the patient’s positive response to therapy, in addition to which she received prednisone, in order to prevent side effects. Viscuse et al. [105] conducted survival analyses to assess the efficacy of first-line ADT versus first-line conventional cytotoxic chemotherapy. The efficacy of salvage ADT in patients treated for recurrent/metastatic SDC or high-grade adenocarcinoma not otherwise specified (NOS) was also evaluated. Abiraterone was used as ADT. The authors concluded that overall survival for first-line ADT and first-line cytotoxic chemotherapy was comparable, but response rates with first-line ADT were higher than those with first-line chemotherapy.

##### COVID-19

The coronavirus pandemic, which has spread around the world in a short period of time, has led to a search for a potential drug or therapy to alleviate symptoms. In a pandemic situation, the reuse of drugs is one of the effective strategies when developing a new therapy. Some studies suggest that androgens play an important role in the entry of SARS-CoV-2, the coronavirus that causes COVID-19, into cells. Namely, there are studies on the fact that patients who underwent ADT for the treatment of prostate cancer suffered from a milder form of COVID-19. Preliminary research suggests that high levels of androgens may increase the risk of severe infection and death from COVID-19 [106,107], although recent research does not support this assumption [108,109].

To find available antiviral drugs against COVID-19, Yuan et al. [110] combined a SARS-CoV-2 enzyme-linked immunosorbent assay and a cell viability assay in their dual screening and applied it to a library consisting of 1528 FDA-approved drugs. Of these, 19 primary emergency compounds showed suitable properties and were subjected to a viral load reduction assay for prioritization based on their dose-dependent anti-SARS-CoV-2 effects. Four compounds were identified as the most potent against SARS-CoV-2, including AA. These four potential drugs were further subjected to in silico testing. AA showed better properties than hydroxychloroquine in docking studies, where AA’s binding free energies were stronger than those of hydroxychloroquine, and additionally, AA formed more stable complexes with the main SARS-CoV-2 protease and the ACE2 receptor [111]. In further in silico studies, Kim et al. [112] studied RNA polymerase and viral proteases. AA led to the inhibition of the production of N proteins and S proteins, and thus to a reduction in viral replication in Vero E6 cells infected with SARS-CoV-2. While RNA-dependent RNA polymerase (RdRp) has been discovered to play a pivotal role in viral replication, Shahabadi et al. [113] have studied four FDA-approved drugs against the RdRp by molecular modeling, docking, and dynamic simulation. Their finding indicated that the selected drugs, including abiraterone, have a high potential to be developed as RdRp inhibitors for the treatment of COVID-19.

Given the controversy surrounding the arrival of COVID-19 vaccines, various studies have emerged on the safety and effectiveness of vaccines, especially among vulnerable cancer populations. The first report of an immune response induced by vaccination against COVID-19 in prostate cancer patients receiving new hormonal treatments (such as AA) confirms that vaccination against COVID-19 was as safe as in matched healthy volunteers [114].

#### 4.1.2. Metabolism of Abiraterone Acetate

After oral administration in capsule form, AA is hydrolyzed to the active metabolite abiraterone, where the conversion is probably mediated by esterase activity. The two main inactive circulating metabolites of abiraterone in human plasma are abiraterone sulfate and abiraterone sulfate *N*-oxide, the formation of which involves the enzymes CYP3A4 and SULT2A1, respectively. The major compounds present in feces are unchanged AA and abiraterone, approximately 55% and 22% of the administered dose, respectively [115].

Li et al. [116] proved that the 3β-hydroxyl structure of abiraterone is subjected to enzymatic conversion by the 3β-hydroxysteroid dehydrogenase (3βHSD) isoenzyme to its 3-keto derivative. The authors confirmed that in mice and patients with prostate cancer, abiraterone is converted to the more active Δ^4^-abiraterone (D4A), which inhibits CYP17A1, 3βHSD, and steroid-5α-reductase (SRD5A), required for DHT synthesis, and then antagonizes the androgen receptor, providing an additional explanation for the clinical abiraterone activity. D4A also has more potent antitumor activity against xenograft tumors than abiraterone. D4A has a 3-keto-4-ene system, which makes the steroid A and B rings identical to testosterone, allowing inhibitory interactions with the AR and additional steroidogenic enzymes.

#### 4.1.3. Side Effects

Abiraterone shows better in vitro selectivity profiles compared to ketoconazole, the first CYP17A1 inhibitor used in the treatment of prostate cancer. However, some serious side effects have been observed in its large clinical trials, due to the interference with other enzymes or receptors. Yin et al. [117] used the term “promiscuous” drug for abiraterone, as it interacts with numerous targets, which dictates its clinical benefits and side effect profile. In addition to inhibiting CYP17A1, abiraterone acts as an androgen receptor antagonist and inhibits 3β-hydroxysteroid dehydrogenase—two effects that potentially contribute to its antitumor effects. Inhibition of 3β-hydroxysteroid dehydrogenase further reduces testosterone production, which may improve the medicinal effects of abiraterone. However, inhibition of the 17α-hydroxylase activity of CYP17A1, CYP11B1, and a panel of hepatic CYP enzymes (including CYP1A2, CYP2D6, CYP2C8, CYP2B6, CYP2C9, CYP2C19, and CYP3A4 and CYP3A5), lead to toxic effects, secondary mineralocorticoid excess, and liver dysfunction, while abiraterone is also associated with an increased incidence of cardiac disorders. The increased cardiovascular risk associated with abiraterone is likely due to the concomitant use of prednisolone/prednisone with abiraterone [54,117,118,119].

Given that abiraterone is an inhibitor of CYP17A1 enzyme activity, and both hydroxylase and lyase, its inhibition leads to the reduced production of 17-hydroxylated pregnenolone and progesterone, and further suppression of lyase activity leads to the reduced production of androgens, which are responsible for the proliferation of cancer cells. So, the side effects are reflected in the increased secretion of pregnenolone and progesterone, which leads to an increase in the level of 11-deoxycorticosterone and the appearance of hypokalemia, hypertension, fluid overload, and the suppression of rennin. On the other hand, the decreased production of cortisol sends feedback and there is an increase in the secretion of ACTH and further accumulation of pregnenolone and progesterone (Figure 7). The addition of dexamethasone to AA results in the suppression of ACTH, the consequent reduction in deoxycorticosterone levels, and the consequent reduction in corticosterone. Similarly, 11-deoxycortisol levels decrease. Inhibition of 17,20-lyase results in redirection to androgen synthesis via the backdoor pathway (purple boxes in Figure 7). AA in combination with dexamethasone, which reverses toxicity and can further reduce androgen levels by preventing an increase in backdoor androgen synthesis, as well as with the selective mineralocorticoid receptor antagonist eplerenone, which affects the control of hypertension and hypokalemia, is a frequently used prostate cancer therapy [52,120,121,122]. However, long-term use of synthetic glucocorticoids is not a therapy that can be tolerated indefinitely, and the search for adequate therapy continues.

### 4.2. Galeterone (***3***)

The constant need for more efficient PC anticancer drugs with fewer side effects led to a shift in the research of anticancer veterans Brodie and Njar from breast cancer to PC [123]. Since CYP17A1 inhibitors have shown to be antagonists of the AR and AR-degrading agents, these authors have performed pharmacophore modeling of human CYP17A1 inhibitors and in vitro inhibition testing on the library of compounds, identifying candidates under code VN/124-1 as a potential anticancer agent [35]. Along with the synthesis, they have reported extensive biological results, identifying VN/124-1, later called galeterone, as a more potent CYP17A1 inhibitor than abiraterone (IC_50_ 300 nM compared to 800 nM for abiraterone) [9]. It has also been shown to be a potent pure AR antagonist that prevents the binding of synthetic androgen methyltrienolone to both the mutant prostate adenocarcinoma LNCaP AR and the wild-type AR. Furthermore, galeterone in vitro inhibited the growth of DHT-stimulated LNCaP and LAPC4 prostate cancer cells, while galeterone in vivo inhibited the growth of androgen-dependent LAPC4 human prostate tumor xenografts [9,124]. Its 50 mg/kg/twice daily application resulted in a 93.8% reduction in the mean final tumor volume compared with controls, making it the first antihormonal agent significantly more efficient than castration in the suppression of androgen-dependent prostate tumor growth. Finally, the same research team reported its in vivo pharmacokinetics testing performed on male mice. After 50 mg/kg galeterone administration, the plasma concentration reached its peak of 16.82 ng/mL within 30 min, and it was no longer detectable after 8 h. 17-(1*H*-Benzimidazol-1-yl)androst-3-one was identified as a galeterone metabolite [9]. These data enabled galeterone to be licensed to Tokai Pharmaceuticals Inc., Cambridge, MA, for further preclinical and clinical studies and granted it a new code name, TOK-001 [123]. After they determined galeterone’s pharmaceutical properties and safety, the FDA granted it Investigation New Drug (IND) status in 2009. The clinical trial program was named ARMOR (androgen receptor modulation optimized for response). In phase 1 trials, ARMOR1, galeterone was administered orally as a powder in a capsule [125]. Phase 1 clinical trials for the treatment of nonmetastatic and metastatic castration-resistant PC indicate that galeterone is well tolerated without the need for prednisone co-administration and with reversible and manageable adverse events (fatigue, aspartate aminotransferase increase, alanine aminotransferase increase, nausea, diarrhea, and pruritus) [126]. Significant prostate-specific antigen and tumor reductions were observed, setting the ground for phase 2 clinical trials. Due to poor oral bioavailability, galeterone was reformulated into a spray-dried dispersion formulation, and the ARMOR2 clinical trial was launched in 2012 [127]. Phase 2 consisted of two parts: part 1 was dedicated to confirming the dose and target patient population, while part 2 worked on the expansion of the dose and patient population selected in part 1. ARMOR2 determined the daily dose to be 2550 mg, confirming the results regarding prostate-specific antigen (PSA) reductions and safety [128]. It also evaluated the PSA responses to subsequent therapy using known drugs in patients previously treated with galeterone. While most PSA responses were modest, the abiraterone response rate was comparable with response rates in patients with no prior galeterone exposure [129]. Phase 3 (ARMOR3-SV) lasted from June 2015 to November 2016, and it was a study of galeterone compared to enzalutamide in men expressing androgen receptor spice variant-7 mRNA (AR-V7) in metastatic castration-resistant prostate cancer [130,131]. Both ARMOR2 and ARMOR3-SV were discontinued in 2016 since Tokai Pharmaceuticals Inc. concluded that trials would likely not succeed in meeting its primary endpoint. This happened because, during phase 3, it was not possible to determine efficacy, since too many patients had advanced cancer before the required radiographs and had to be taken off the trial [132]. However, in 2018, the University of Maryland, Baltimore, granted Educational and Scientific LLC (ESL) an exclusive license for galeterone development for the treatment of castration-resistant PC [133]. The results of these studies are yet to be published, but galeterone biological activities are being extensively tested and continue to trigger scientific curiosity.

#### 4.2.1. Enzyme Inhibition

The anticancer activity of galeterone is partially due to its CYP17 inhibitor activity. Galeterone selectively and irreversibly inhibits CYP17A1 and prevents intratumoral androgen synthesis. By selectively inhibiting 17,20 lyase activity of CYP17A1, galeterone decreases the DHEA and androstenedione production, while slightly increasing gene expression of HSD3B2, a gene involved in androgen biosynthesis [134]. It does not inhibit aromatase (CYP19A1) [134], while the results of the 21-hydroxylase activity of steroid 21-monooxygenase (CYP21A2) are conflicting. Some reports indicate that galeterone does not inhibit this enzyme [134], while others claim that it inhibits CYP21A2 much better than its pharmacologically active metabolite, 3-keto-Δ^4^-galeterone, which can be considered inactive [135]. There is also a study showing that galeterone specifically inhibits USP12 and USP46, two highly homologous deubiquitinating enzymes [136]. Both of these enzymes can control AR signaling, but herein, through inhibiting USP12 and USP46 activity, galeterone was able to inhibit cell growth even in AR-negative PC.

#### 4.2.2. Interaction with Androgen Receptors

One of the proposed mechanisms of galeterone action is based on its interaction with androgen receptors. It was determined that galeterone causes in vitro and in vivo down-regulation of AR protein expression, unlike clinically used antiandrogen bicalutamide, and castration that caused significant up-regulation of AR protein expression [137]. Galeterone is a direct and competitive AR antagonist, as well as a progesterone receptor antagonist [138], and prevents AR from binding to chromatin, while in low micromolar concentrations, it enhances the degradation of mutant AR [139]. Furthermore, the galeterone mechanism of action is, among others, based on disrupting AR signaling via a proteasomal-dependent pathway, leading to AR degradation [140]. It is also reported that apoptosis in PC cells induced by galeterone was due to increased Bax/Bcl2 ratios and cytochrome-c release with concomitant cleavage of caspase 3 and PARP cleavage [140].

#### 4.2.3. Other Cancers

The mRNA 5′ cap-binding protein eukaryotic translation initiation factor 4E (eIF4E) is a pivotal factor that initiates translation, a rate-limiting step in protein synthesis. By down-regulating protein synthesis, oncogenesis and cancer progression can be controlled. Inhibition of eIF4E phosphorylation is one of the newest approaches in cancer control. Since phosphorylation is performed by the mitogen-activated protein kinase-interacting kinases (MNKs, including MNK1 and MNK2), the MNK-eIF4E axis represents a therapeutic target for novel anticancer drugs [141]. The first testing of this type of galeterone activity was performed regarding prostate cancer [142], but these results have also allowed to broaden research scope to other types of cancer, such as pancreatic [143] and breast [144]. Galeterone targets a couple of epithelial-to-mesenchymal transition (EMT) markers (Snail, Slug, N-cadherin, vimentin, and MMP-2/-9) via antagonizing the Mnk–eIF4E axis, which results in inhibition of migration and invasion of prostate cancer cells [142]. Besides this activity, in pancreatic ductal adenocarcinoma (PDAC), galeterone strongly inhibited MiaPaca2 tumor xenograft growth and cell viability of gemcitabine-naïve/-resistant PDAC cell lines and strongly synergized with gemcitabine in gemcitabine-resistant PDAC cells [143]. The last one represents an important finding since gemcitabine is the standard chemotherapeutic drug for locally advanced and metastatic PDAC. Suppressing MNK-eIF4E and β-catenin was identified as a mechanism of galeterone action on breast cancer cells [144]. Galeterone decreased the proliferation of breast cancer cell lines MDAMB-231, MDA-MB-68, Hs 578T, and BT-549, with IC_50_ values ranging from 0.5 to 4 μM, all due to inhibiting the MNK-eIF4E axis.

#### 4.2.4. Metabolism of Galeterone

**Scheme 9:**
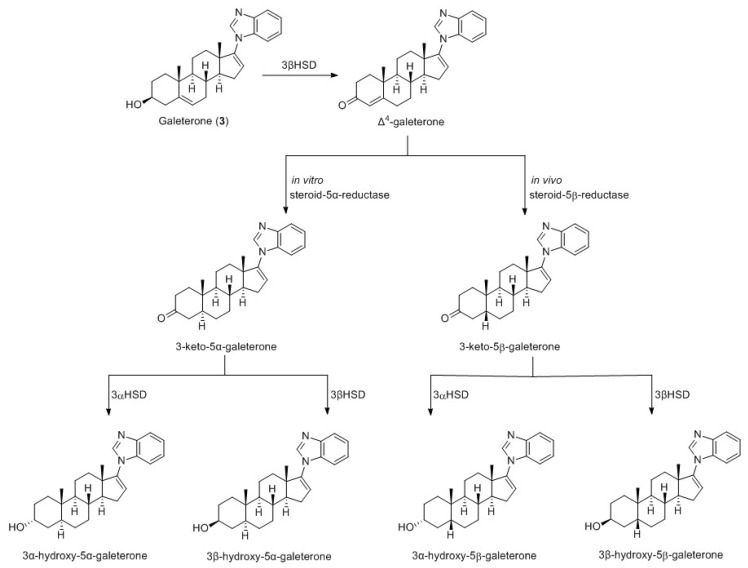
In vitro and in vivo metabolism of galeterone.

The metabolism of drug candidates influences both their activity and toxicity and thus represents an important step in biological studies. Due to its steroidal structure, galeterone is metabolized by steroidogenic enzymes because it mimics their natural ligands [145]. In vitro and in vivo studies on rodents showed that galeterone metabolism starts with dehydrogenation by 3βHSD to Δ^4^-galeterone, which is further converted in vitro to 3-keto-5α-galeterone, 3α-hydroxy-5α-galeterone, and 3β-hydroxy-5α-galeterone by the action of steroid-5α-reductase, while it is converted in vivo to corresponding 5β-reduced metabolites (Scheme 9) [146]. Δ^4^-galeterone inhibits enzymes and suppresses AR protein stability, AR target gene expression, and xenograft growth comparably with galeterone. Unfortunately, the second metabolic step leads to the loss of several activities that inhibit the androgen axis. This is probably responsible for lower clinical efficacy compared to abiraterone.

#### 4.2.5. Galeterone Formulation

Galeterone administration during phase 1 was orally in the form of a powder in a capsule, but to increase its bioavailability for phase 2, it was formulated as a 50–50 (*w*/*w*) galeterone–hypromellose acetate succinate spray-dried dispersion [147]. Since both phases 2 and 3 were discontinued due to efficacy problems, the dilemma still stands as to whether metabolism is the responsible factor or if the formulation plays a part in the decrease in activity. A recent study was performed in order to create a new galeterone formulation as an amorphous solid dispersion by KinetiSol^®^ compounding [148]. A comparison of KinetiSol and spray drying showed an approximate six-time increase in dissolution performance and a two-time better exposure in an in vivo rat study with KinetiSol. The KinetiSol amorphous solid dispersion formulation was better at protecting galeterone with weak basic properties from premature dissolution in acidic media. This has reduced precipitation, inhibited recrystallization, and extended supersaturation during transit into neutral intestinal media, increasing overall oral bioavailability.

### 4.3. Comparative Studies of Biological Activity of Abiraterone and Galeterone

Although abiraterone is an efficient anticancer drug, a search for more selective and active medicines has led to the development of the drug candidate galeterone. Their structural similarities led to the inevitable comparison of biological activities. Cell-based assays were utilized and confirmed that abiraterone and galeterone inhibit nuclear translocation of sterol-regulatory element binding protein 2 and the transcription of mevalonate genes, indicating their potential against castration-resistant PC [149]. Inhibition of androgen-independent human prostate cancer cell line (CWR22Rv1) proliferation was tested using an MTT assay, where galeterone was more potent than abiraterone, with GI_50_ values of 3.8 and 11.46 μM, respectively [39]. When their antitumor efficacy was compared in vivo using the LAPC4 human prostate cancer xenograft model, galeterone again showed higher activity than abiraterone, as well as AA [150]. The mechanism of these actions consists of inhibition of steroidogenic enzymes or interaction with androgen receptors. Both galeterone and abiraterone reduce AR protein and mRNA expression [151]. Galeterone is superior to abiraterone since it is an effective apparent competitor for binding to the wild type and various mutant AR (W741C, W741L) proteins. On the other hand, the first-in-class CYP17A1 inhibitor abiraterone demonstrated a three-fold higher inhibition of 17,20-lyase activity than galeterone but later had three times higher selectivity towards 17,20-lyase inhibition over hydroxylase inhibition [152]. These molecules showed similar inhibition of human liver cytosolic DHEA sulfonation, in the same study that confirmed the prodrug activity of abiraterone acetate [153]. Analysis of abiraterone and galeterone interaction with human sterol 14α-demethylase (cytochrome P450 51A1, CYP51A1) did not identify inhibition but did identify monohydroxylation of both compounds [154].

These results indicated that galeterone would pass through clinical studies and show better efficacy than abiraterone, but unfortunately, it did not, probably for the reasons stated in previous sections: galeterone metabolism or its formulation, or the problem is more of a technical nature and refers to the implementation of clinical studies. This is yet to be determined but will be clearer when the results of the last phase of clinical studies performed by ESL are published.

## 5. Biologically Active Derivatives and Analogs of Galeterone and Abiraterone

Galeterone and abiraterone are structural motifs for the molecular design of a vast majority of novel molecules with potential biological activity. In this section, new potential therapeutics for prostate cancer are described, which, according to their structural characteristics, belong to galeterone and abiraterone derivatives, focusing on agents targeting PC in preclinical development.

**Figure 8:**
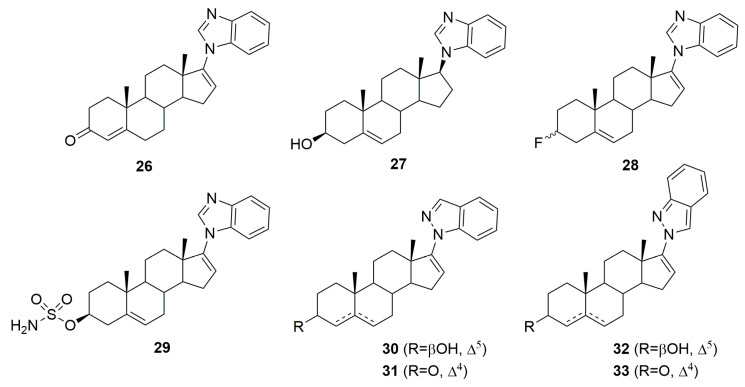
Galeterone derivatives **26**–**33**.

One of the first reported analogs, synthesized along with galeterone, was its Δ^4^-3-keto derivative **26** which also showed dual activity as a potent CYP17A1 inhibitor and antagonist of AR (Figure 8) [9]. In the next few years, intensive studies were carried out during which putative metabolites of galeterone, including Δ^16^-saturated **27**, as well as metabolically stable derivatives such as 3-fluoro and 3-*O*-sulfamoyl derivatives (Figure 8, compounds **28** and **29**), were prepared [150]. In vitro studies, including CYP17A1 inhibitory activity, binding to AR, and antiproliferative effects against LNCaP and LAPC4 cell lines showed that none of these compounds was more potent than galeterone. However, in vivo studies revealed that 3-fluoro derivative **28** was almost 2-fold more active against LAPC4 than galeterone, but unfortunately, toxicity was observed with this halogenated compound [150]. The good antitumor activity of galeterone is due to the presence of the benzimidazole ring, which was the inspiration for the design and synthesis of new steroid inhibitors containing *N*-heterocyclic groups related to benzimidazole. Several 1*H*- and 2*H*-indazole derivatives of dehydroepiandrosterone acetate (compounds **30**–**33**, Figure 8) were tested for human CYP17A1 inhibition, androgen receptor (AR) binding affinity, and cytotoxicity against three PC cell lines. The compounds did not significantly inhibit C_17,20_-lyase or show affinity for LNCaP-mutated AR [155]; however, the 2*H*-indazole series exhibited significant cytotoxicity against AR-negative PC-3 cells [156].

**Figure 9:**
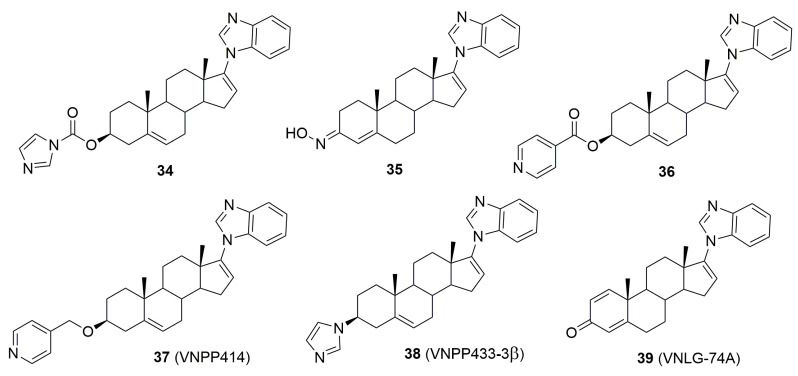
C-3-modified derivatives of galeterone.

In order to evaluate the influence of small structural modifications of galeterone on the modulation of the AR, Purushottamachar et al. [157] synthesized and tested a series of C-3, C-16, and C-17 analogs. Based on the SAR analysis, they concluded that the benzimidazole ring at C-17 is of greatest importance for biological activity, as well as that the polar and heteroaromatic groups at C-3 promote both antiproliferative activity and the degradation of androgen receptors. Therefore, the most potent antiproliferative activity showed carbamate derivative **34**, hydoxyimino derivative **35**, and pyridin-4-yl analog **36** (Figure 9), with GI_50_ values of 0.87, 1.91, and 2.57 μM, respectively. Compared to galeterone, compound **34** was 4- and 8-fold more potent with respect to antiproliferative and AR-degrading activities, respectively [157]. Further antitumor efficacy studies showed that **34** was moderately more effective than galeterone (70 versus 60% growth inhibition, respectively, compared to control) in inhibiting the growth of castration-resistant prostate cancer CWR22Rv1 (androgen-independent human prostate cancer cell line) tumor xenografts [140]. Nevertheless, the presence of metabolically labile functions such as carbamate and ester groups in compounds **34** and **36**, respectively, remained a concern.

In continuation of their research, Purushottamachar et al. [158] prepared and investigated new C-3-modified analogs of galeterone. Out of a total of 11 tested compounds, 3β-pyridyl ether **37** and 3β-imidazole derivative **38** (Figure 9), with antiproliferative activity GI_50_ of 3.24 and 2.54 μM against CWR22Rv1 prostate cancer cells, were 2.75 and 3.5 times more active than galeterone. These two leading analogs of galeterone, compounds **37** (VNPP414) and **38** (VNPP433-3β), were further investigated using in vitro and in vivo prostate cancer (PC) models. Selected derivatives have been shown to degrade AR/AR-V7 and Mnk1/2 (mitogen-activated protein kinases 1 and 2), and then block human PC cell proliferation, induce apoptosis, inhibit cell migration, etc. Additionally, galeterone and its analogs (alone or in combination) reduced the growth of human PC cell lines resistant to certain antineoplastic drugs. During in vivo testing, VNPP433-3β significantly reduced the growth of castration-resistant xenograft (CRPC) CWR22Rv1 tumors, without significant toxicity [159]. Recently, VNPP433-3β was found to inhibit cancer stem cells (CSCs) in prostate cancer, possibly by degrading AR. Transcriptome analyses show that VNPP433-3β blocks the transcription of several genes and functional pathways crucial for prostate CSCs [160]. Regarding the mechanism of action, VNPP433-3β exerts its antitumor effect through transcriptional regulation of AR and AR-responsive oncogenes, regulation of translation by interfering with mRNA-5′ cap-dependent translation initiation, and reduction in AR half-life [161]. Given the highly potent anticancer efficacy of VNPP433-3β, the obtained murine toxicology and pharmacokinetic profiles indicated that the compound could be further developed as a potential anticancer drug [162]. In addition to all the previous tests of the next generation galeterone analog VNPP433-3β, by using HiBiT (11 amino acid peptide) CRISPR (clustered regularly interspaced short palindromic repeats) cell lines, biochemical methods, and RNA sequencing, Thomas et al. [163] reported the potential role of the compound as molecular glue. In this way, it brings together AR, the key driver of prostate cancer, and MDM2 (mouse double minute 2 gene), an E3 ubiquitin ligase leading to ubiquitination and subsequent degradation of f-AR and AR-V7 in prostate cancer cells.

Apart from the exhaustive preclinical investigation, the same group of authors has also described a large multi-gram scale synthesis of compound **38** (VNPP433-3β) starting from the commercially available dehydroepiandrosterone 3-acetate (DHEA) via galeterone, in eight steps with a 26% overall yield and 99.5% purity. The main advantages of this method are reflected in the fact that they managed to avoid column purification in several synthetic steps and reduce the consumption of expensive and toxic solvents and reagents by using alternative reagents [39].

Two previously mentioned galeterone analogs, **36** and **39** (VNLG-74A) (Figure 9), were tested in PC cell models of different androgen and AR dependences. McCarthy et al. [164] concluded that both derivatives possess better antiproliferative properties and have higher antiandrogenic activity compared to abiraterone and enzalutamide. They predicted, from in silico molecular modeling, that **36** and **39** bind to the ATPase domain of BiP/Grp78 and Hsp70-1A with higher affinity than AR, suggesting that disruption of the function of the 70 kDa heat shock protein is a likely mechanism of action for these analogs.

**Figure 10:**
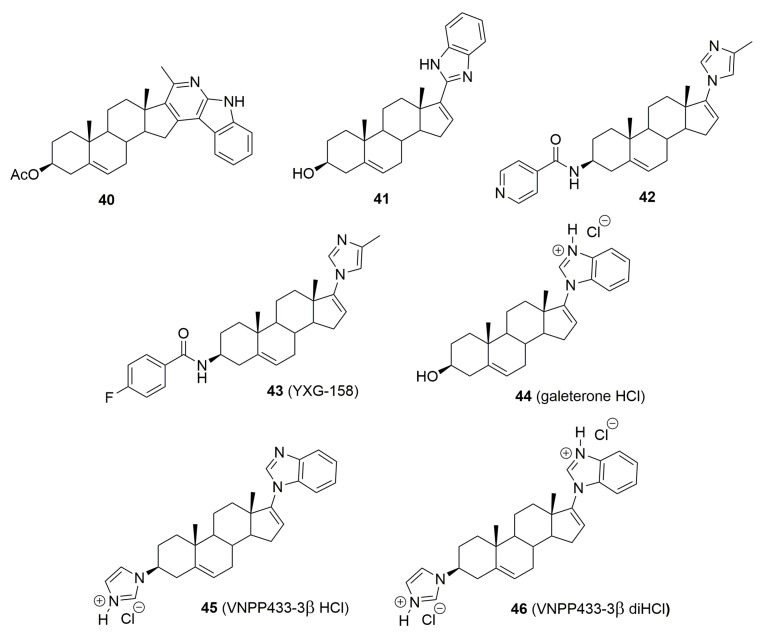
Structures of galeterone analogs **40**–**46**.

Jorda and co-workers [165] tested, against PC cell lines, a series of galeterone analogs, including several steroid-condensed azacycles, as well as 17-(benzimidazol-1-ylimino), 16α-(benzimidazol-2-ylamino), and 16α-(benzothiazol)-2-ylamino) steroid derivatives. Compound **40** (Figure 10) was found to exhibit down-regulation of AR-regulated transcription and suppressed expression of AR-regulated proteins in androgen-dependent prostate cancer cells, 22Rv1-ARE14 and VCaP. In silico analysis showed that compound **40** binds to the AR in a similar manner to that of galeterone, even with a higher binding energy.

In their work, Latysheva et al. [166] synthesized several new steroidal derivatives related to abiraterone and galeterone, where two of them potently inhibited the growth of prostate carcinoma cells, LNCaP and PC-3. Promising derivative **41** (Figure 10), with a GI_50_ of 3.8 and 6.9 μM, is designed as a structural isomer of galeterone, in which the benzimidazole moiety is linked to the steroid by forming a C2′-C17 bond rather than the N1′-C17 bond found in galeterone.

Recently described structural modifications on the C-3, C-6, and C-17 positions of galeterone led to the discovery of compound **42** (Figure 10) with the dual functions of AR antagonism and degradation [167]. The amide derivative **42** was shown to be a potent antiproliferative agent with the ability to degrade AR in various PCa cells (LNCaP and 22RV1), with antagonistic activity against wild-type and mutant ARs. In in vivo experiments, compound **42** reduced the growth of hormone-sensitive organs and showed tumor regression in an enzalutamide-resistant (c4-2b-ENZ) xenograft model. Further research on 17-imidazole analogs of galeterone revealed new selective AR degraders, among which **43** (YXG-158, Figure 10) was the most potent antitumor compound with dual functions of AR degradation and CYP17A1 inhibition [168]. Additionally, **43** effectively inhibited the growth of hormone-sensitive organs such as **42** and exhibited robust antitumor efficacy both in enzalutamide-sensitive (LNCaP/AR) and enzalutamide-resistant (C4-2b-ENZ) xenograft models. Therefore, compound **43** was chosen as a preclinical candidate for the treatment of enzalutamide-resistant prostate cancer.

The latest research has identified new excellent orally bioavailable candidates for clinical development, the monohydrochloride salt of galeterone **44** and the mono- and di-hydrochlorides salts of VNPP433-3β (**38**), referred to as compounds **45** and **46**, respectively (Figure 10) [169]. It was found that compound **44** displayed enhanced in vitro antiproliferative activity against three different PC cell lines, LNCaP (androgen-sensitive), C42B (androgen-insensitive), and CWR22Rv1 (castration-resistant), but decreased plasma exposure in the pharmacokinetics study. The antiproliferative activity of salts **45** and **46** was equivalent to that of compound **38**, but their oral pharmacokinetic profile was significantly enhanced. Finally, oral administration of the parent compounds (galeterone and VNPP433-3β) and their corresponding salts, **44**, **45**, and **46**, caused dose-dependent potent inhibition/regression of aggressive CWR22Rv1 tumor xenograft growth, without host toxicities.

**Figure 11:**
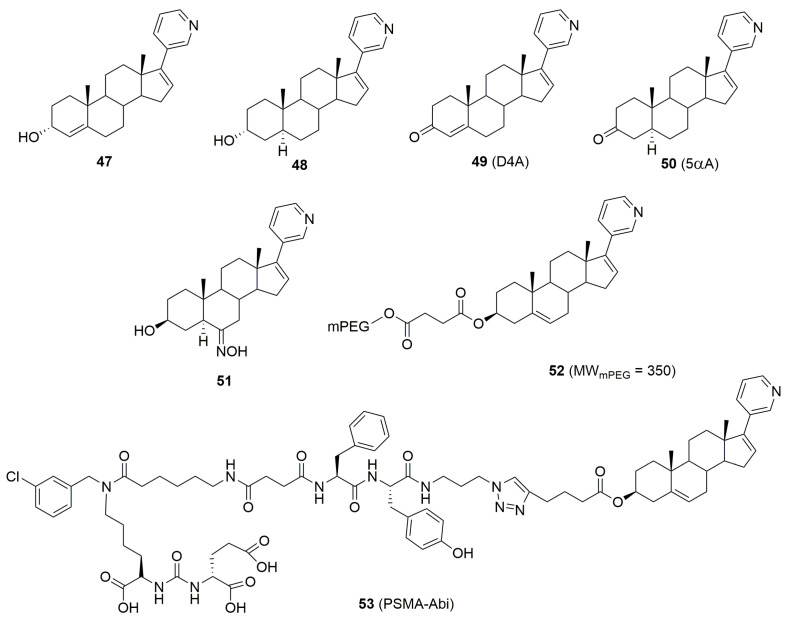
Synthesized abiraterone analogs **47**–**53**.

As for abiraterone, far fewer derivatives and analogs have been synthesized and tested compared to galeterone. One of the first reports was published by Garrido and co-workers [170] who synthesized four analogs of abiraterone: 3α-hydroxy-Δ^4^-abiraterone (**47**), 3α-hydroxy-5α-abiraterone (**48**), and two abiraterone metabolites, D4A (**49**) and 3-keto-5α-abiraterone (**50**, 5αA) (Figure 11). For these analogs and for ketoconazole, the spectral binding constants (Ks) were measured using purified and modified human CYP17A1, along with the determination constants (Ki) applying a native human CYP17A1 enzyme in yeast microsomes. The authors concluded that the three abiraterone-related steroidal azoles, **48**, **49**, and **50**, which also mimic natural substrates, appeared to be extraordinarily potent inhibitors of human CYP17A1, whereas **47** is moderately potent and comparable to ketoconazole.

One of the main side effects of D4A, a more pharmacologically active abiraterone metabolite, may be the disruption of corticosteroid biosynthesis. Masamrekh et al. [171] therefore evaluated both the in silico and in vitro inhibitory activity of D4A (**49**) on one of the key corticosteroidogenic enzymes, human steroid 21-monooxygenase (CYP21A2). It was found that D4A demonstrates effects similar to those of abiraterone on CYP21A2, which indicates that it might disrupt gluco- and mineralocorticoid metabolism.

Fehl et al. [172] have designed abiraterone analogs based on structural evidence that B-ring substituents may favorably interact with polar residues when binding CYP17A1 and sterically clash with residues in the CYP21A2 active site. The best analog, **51** (Figure 11), increased the selectivity of CYP17A1 inhibition 84-fold compared with 6.6-fold for abiraterone. Co-crystallization with CYP17A1 confirmed the new predicted contacts with CYP17A1 active site residues. A molecular docking study of this analog on CYP21A2 revealed steric clashes that likely underlie decreased binding and CYP21A2 inhibition.

The latest research on abiraterone derivatives as prostate anticancer agents is based on the design and synthesis of a series of compounds with methoxypoly(ethylene glycol) (mPEG) modification [173]. Their antitumor activities against three PC cell lines (DU145, LNCaP, and PC3) and toxicology were analyzed in vitro and in vivo, where the most potent compound, **52** (Figure 11), significantly improved the abiraterone water solubility, cell permeability, and blood safety. It was observed that mPEG modification significantly improved abiraterone’s antitumor activity and efficiency while reducing the associated toxic effects.

In their study, Machulkin et al. [174] synthesized a new PSMA-targeted conjugate based on abiraterone (**53**, PSMA-Abi, Figure 11) and investigated its cytotoxicity, the induction of intracellular reactive oxygen species, and P450-cytochrome species inhibition. The conjugate showed a significant effect on PC-3 and 22Rv1 prostate tumor cells (CC_50_ 8.1 and 8.2 μM, respectively). This compound was comparable in efficacy to abiraterone acetate, with significantly reduced acute toxicity.

**Figure 12:**
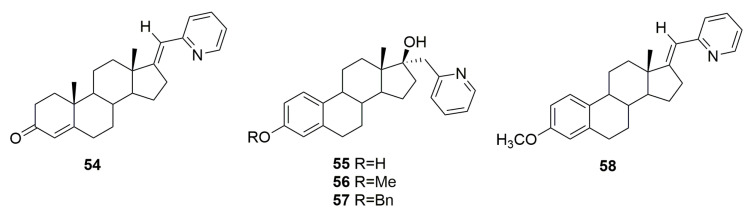
Synthesized abiraterone analogs **55**–**58**.

Given the importance of the topic and the need for the development of new agents in the fight against prostate cancer, our research group also dealt with the synthesis of abiraterone analogs and tested their antitumor activity. Thus, using the radiosubstrate in vitro incubation methods for the determination of the distinct 17α-hydroxylase and C_17,20_-lyase activities of the CYP17A1 enzyme, we have studied the inhibitory activity of selected steroidal 17α-picolyl and (17*E*)-picolinylidene compounds. The tests revealed a substantial inhibitory action of (17*E*)-(pyridin-2-ylmethylidene)androst-4-en-3-one (**54**, Figure 12) towards both the 17α-hydroxylase and C_17,20_-lyase activities of the CYP17A1 [175]. A similar group of abiraterone analogs, **55**–**58**, with 17α-picolyl or (17*E*)-picolinylidene groups (Figure 12), only in the estrane series, was shown to be active against PC3 prostate cancer cells, with IC_50_ values ranging from 4.69 to 8.44 µM. They also showed high selectivity and superiority over doxorubicin [176].

## 6. Conclusions

The therapeutic significance of abiraterone, especially in the treatment of castration-resistant prostate cancer, led to the publication of a large number of scientific results which, besides its biological activity, include multiple methods of abiraterone synthesis, as well as the development of novel steroid derivatives modeled by its structure. Among those, galeterone stands out for its bioactivity, and this made it progress the furthest in clinical studies. Its mechanism of action is important for further drug design, and it is mostly based on the inhibition of androgen production. Selective inhibition of C_17,20_-lyase activity of CYP17A1 can completely block androgen production, while inhibition of 17α-hydroxylase activity leads to the accumulation of pregnenolone and progesterone and, consequently, the overproduction of mineralocorticoids. Abiraterone inhibits both, and for this reason, the search for new drugs that would exclusively block the lyase activity of the CYP17A1 enzyme continues; as a result of this research, galeterone was synthesized. Galeterone acts by a trimodal mechanism: CYP17A1 inhibition, competitive inhibition, and down-regulation of the androgen receptors; however, probably due to its metabolism, it shows lower clinical efficacy. But it must be pointed out that galeterone has three times higher selectivity towards 17,20-lyase inhibition over hydroxylase inhibition. This clinical efficacy of galeterone compared with abiraterone leads to a continuation of the hunt for selective 17,20-lyase inhibitors.
